# Redetermination of di-μ-sulfido-bis­{[(2*R*)-2-acet­oxy-2-amino­ethane-1-thiol­ato-κ^2^ *N*,*S*]oxidomolybdenum(V)}

**DOI:** 10.1107/S1600536810014625

**Published:** 2010-04-24

**Authors:** Haruo Akashi, Yuji Shiraga, Takashi Shibahara

**Affiliations:** a Research Institute of Natural Sciences, Okayama University of Science, Ridai-cho, kita-ku, Okayama 700-0005, Japan; b Department of Chemistry, Okayama University of Science, Ridai-cho, Kita-ku, Okayama 700-0005, Japan

## Abstract

The structure of the title compound, [Mo_2_(C_4_H_8_NO_2_S)_2_O_2_S_2_], has been redetermined. Besides obvious differences between the original [Drew & Kay (1971 ▶). *J. Chem. Soc. A*, pp. 1851–1854] and the current unit-cell parameters, some packing features of the structure are also different; these findings are the result of significant improvements in the precision and accuracy of the present structure analysis. The two Mo atoms in the dimeric complex have very similar distorted trigonal–bipyramidal environments. Each Mo atom is bonded to an S atom and to an N atom of an l-cysteine ester ligand, to a terminal O atom and to two S atoms which bridge to the adjacent Mo atom [Mo⋯Mo separation = 2.8191 (2) Å]. N—H⋯O_carbon­yl_ and N—H⋯O_terminal_ hydrogen-bonding inter­actions consolidate the crystal packing. During the synthesis, the originally employed l-cysteinate ligand has been converted to the l-cysteinate methyl ester ligand. Since this reaction does not take place without tin(IV) chloride, it is clear that tin(IV) chloride acts as a catalyst for the reaction.

## Related literature

For the properties of molybdenum complexes with sulfur ligands, see: Newton & Otsuka (1980 ▶); Ueyama *et al.* (1982 ▶). For syntheses of related compounds, see: Shibahara & Akashi (1992 ▶); Kay & Mitchell (1970 ▶). For related structures, see: Shibahara *et al.* (1983 ▶); Drew & Kay (1971 ▶).
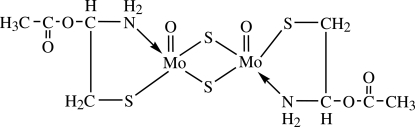


## Experimental

### 

#### Crystal data

[Mo_2_(C_4_H_8_NO_2_S)_2_O_2_S_2_]
*M* *_r_* = 556.34
Monoclinic, 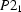

*a* = 9.195 (5) Å
*b* = 5.622 (3) Å
*c* = 17.437 (9) Å
β = 91.6763 (15)°
*V* = 901.0 (8) Å^3^
*Z* = 2
Mo *K*α radiation
μ = 1.88 mm^−1^
*T* = 93 K
0.32 × 0.23 × 0.15 mm

#### Data collection

Rigaku Mercury diffractometer
Absorption correction: multi-scan (REQAB; Jacobson, 1998 ▶) *T*_min_ = 0.680, *T*_max_ = 0.759
10030 measured reflections
5007 independent reflections
4966 reflections with *F* ^2^ > 2σ(*F* ^2^)
*R*_int_ = 0.024

#### Refinement

*R*[*F* ^2^ > 2σ(*F* ^2^)] = 0.024
*wR*(*F* ^2^) = 0.067
*S* = 1.12
5007 reflections
200 parameters
H-atom parameters constrained
Δρ_max_ = 1.23 e Å^−3^
Δρ_min_ = −0.63 e Å^−3^
Absolute structure: Flack (1983 ▶), 2185 Friedel pairs
Flack parameter: −0.08 (3)

### 

Data collection: *CrystalClear* (Rigaku, 1999 ▶); cell refinement: *CrystalClear*; data reduction: *CrystalStructure* (Rigaku Americas and Rigaku, 2007 ▶); program(s) used to solve structure: *SIR2004* (Burla *et al.*, 2005 ▶); program(s) used to refine structure: *SHELXL97* (Sheldrick *et al.*, 2008 ▶); molecular graphics: *ORTEPIII* (Burnett & Johnson, 1996 ▶); software used to prepare material for publication: *CrystalStructure*.

## Supplementary Material

Crystal structure: contains datablocks global, I. DOI: 10.1107/S1600536810014625/wm2325sup1.cif

Structure factors: contains datablocks I. DOI: 10.1107/S1600536810014625/wm2325Isup2.hkl

Additional supplementary materials:  crystallographic information; 3D view; checkCIF report

## Figures and Tables

**Fig. 1.**
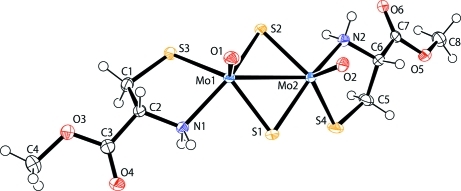
Molecular configuration and atom-numbering scheme for compound (I) with displacement ellipsoids drawn at the 50% probability level.

**Fig. 2.**
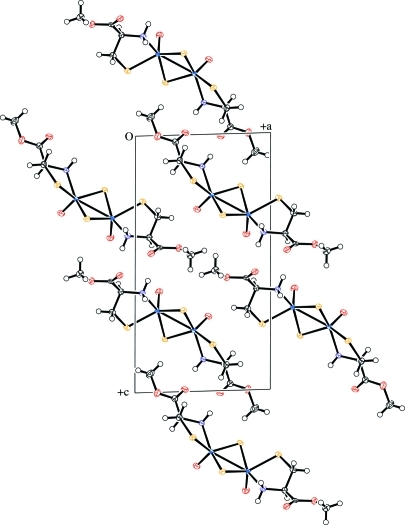
A view of the molecular packing of the structutre of compound (I) along the b axis. H atoms have been omitted for clarity.

**Fig. 3.**
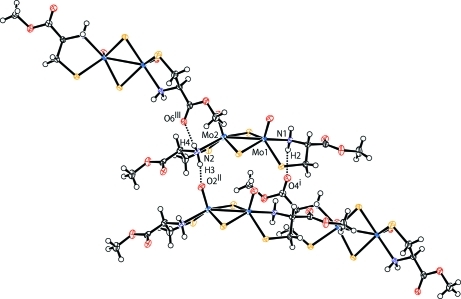
Scheme of intermolecular N—H···O interactions of (I). Symmetry codes: (i) -x+1, y+1/2, -z, (ii) x, y+1, z, (iii) -x+2, y-1/2, -z+1.

## supplementary crystallographic information

### Comment

Molybdenum complexes with sulfur ligands including L-cysteine or L-cysteine ethers are of interest in relation to redox-active molybdo-enzymes (Newton *et al.*, 1980). Doubly sulfur-bridged molybdenum(V) compounds are prepared and examined as catalysts for redox reactions (Ueyama *et al.*, 1982). The formation of the title compound, C_8_H_16_Mo_2_N_2_O_6_S_8_, (I), has been reported previously in the reaction of sodium molybdate with hydrogen sulphide and L-cysteine methyl ester (Kay & Mitchell, 1970). However, the direct formation of (I) from Na_2_[Mo_2_O_2_S_2_(L-cys)_2_] (Shibahara & Akashi, 1992) has not been reported previously. In this reaction in methanol, the L-cysteinato ligand has changed to the L-cysteinato methyl ester ligand.

The structure of (I) has been reported previously by Drew & Kay (1971), but there are significant differences between the original and the current unit cell parameters which, in part, may be ascribed to the different measurement temperatures: Drew & Kay (1971), room temperature measurement: monoclinic, *P*2_1_, with *a* = 9.348 (9), *b* = 5.640 (7), *c* = 19.440 (16) Å, *β* = 116.66 (10)°. This work: monoclinic, *P*2_1_, with *a* = 9.195 (5), *b* = 5.622 (3), *c* = 17.437, *β* = 91.6763 (15)°. In the present work, the structure of (I) (Fig. 1) was determined with sufficient accuracy (R-factor = 0.024) and all hydrogen atoms in the structure were refined. The Mo - Mo distance is 2.8191 (2) Å. The Mo-S_bridge_ distances are 3.079 (7) and 3.3941 (7) Å. The range of these distances is within the range of values observed previously in doubly sulfur-bridged molybdenum(V) compounds, see, for example: Shibahara *et al.* (1983). The packing of the structure of (I) (Fig. 2) is also obviously different from that reported by Drew & Kay (1971). It is clear that *N*—*H*···O_carbonyl_ and N—H···O_terminal_ intermolecular hydrogen bonds exist in the structure of (I) (Fig. 3).

### Experimental

Tin(IV) chloride pentahydrate (108.8 mg, 0.155 mmol) was added to Na_2_[Mo_2_O_2_S_2_(L-cys)_2_] (100 mg, 0.155 mmol) in methanol (40 ml). Single crystals suitable for X-ray diffraction were grown from the solution through slow evaporation of the solvent.

### Refinement

The positions of all H atoms were initially located from difference maps and were refined by using the riding model. The isotropic displacement parameters for these atoms were fixed at 1.2 times the equivalent isotropic displacement parameter of their carrier atom.

### Crystal data

**Table:**

[Mo_2_(C_4_H_8_NO_2_S)_2_O_2_S_2_]	*F*(000) = 548.00
*M**_r_* = 556.34	*D*_x_ = 2.051 Mg m^−^^3^
Monoclinic, *P*2_1_	Mo *K*α radiation, λ = 0.71070 Å
Hall symbol: P 2yb	Cell parameters from 3110 reflections
*a* = 9.195 (5) Å	θ = 5.5–30.0°
*b* = 5.622 (3) Å	µ = 1.88 mm^−^^1^
*c* = 17.437 (9) Å	*T* = 93 K
β = 91.6763 (15)°	Platelet, orange
*V* = 901.0 (8) Å^3^	0.32 × 0.23 × 0.15 mm
*Z* = 2	

### Data collection

**Table:**

Rigaku Mercury diffractometer	4966 reflections with *F*^2^ > 2σ(*F*^2^)
Detector resolution: 7.31 pixels mm^-1^	*R*_int_ = 0.024
ω scans	θ_max_ = 30.0°
Absorption correction: multi-scan (REQAB; Jacobson, 1998)	*h* = −12→12
*T*_min_ = 0.680, *T*_max_ = 0.759	*k* = −7→7
10030 measured reflections	*l* = −24→24
5007 independent reflections	

### Refinement

**Table:**

Refinement on *F*^2^	*w* = 1/[σ^2^(*F*_o_^2^) + (0.0278*P*)^2^ + 0.6607*P*] where *P* = (*F*_o_^2^ + 2*F*_c_^2^)/3
*R*[*F*^2^ > 2σ(*F*^2^)] = 0.024	(Δ/σ)_max_ = 0.007
*wR*(*F*^2^) = 0.067	Δρ_max_ = 1.23 e Å^−^^3^
*S* = 1.12	Δρ_min_ = −0.63 e Å^−^^3^
5007 reflections	Absolute structure: Flack (1983), 2185 Friedel pairs
200 parameters	Flack parameter: −0.08 (3)
H-atom parameters constrained	

### Special details

**Table:**

Refinement. Refinement was performed using all reflections. The weighted *R*-factor (wR) and goodness of fit (*S*) are based on *F*^2^. *R*-factor (gt) are based on *F*. The threshold expression of *F*^2^ > 2.0 σ(*F*^2^) is used only for calculating *R*-factor (gt).

### Fractional atomic coordinates and isotropic or equivalent isotropic displacement parameters (Å^2^)

**Table:**

	*x*	*y*	*z*	*U*_iso_*/*U*_eq_	
Mo(1)	0.56160 (2)	0.35614 (4)	0.237407 (11)	0.01531 (5)	
Mo(2)	0.83145 (2)	0.39138 (4)	0.317580 (11)	0.01609 (5)	
S(1)	0.77619 (7)	0.16995 (14)	0.20725 (4)	0.02024 (13)	
S(2)	0.64491 (7)	0.66478 (13)	0.31650 (4)	0.01814 (13)	
S(3)	0.42535 (7)	0.67618 (13)	0.18011 (4)	0.01799 (12)	
S(4)	1.05984 (8)	0.40575 (19)	0.25678 (4)	0.02909 (17)	
O(1)	0.4563 (2)	0.1814 (4)	0.29126 (11)	0.0214 (4)	
O(2)	0.8209 (2)	0.1968 (4)	0.39132 (12)	0.0229 (4)	
O(3)	0.1638 (2)	0.2314 (5)	0.00266 (13)	0.0307 (5)	
O(4)	0.3678 (2)	0.0163 (4)	−0.00768 (12)	0.0238 (4)	
O(5)	1.3237 (2)	0.8121 (4)	0.44041 (12)	0.0255 (4)	
O(6)	1.1181 (2)	1.0185 (4)	0.45859 (12)	0.0235 (4)	
N(1)	0.4930 (2)	0.2095 (4)	0.12429 (12)	0.0163 (4)	
N(2)	0.9461 (2)	0.6727 (4)	0.38715 (13)	0.0187 (4)	
C(1)	0.3251 (3)	0.5388 (5)	0.09977 (16)	0.0199 (5)	
C(2)	0.3400 (2)	0.2688 (5)	0.10319 (14)	0.0169 (4)	
C(3)	0.2942 (2)	0.1557 (5)	0.02689 (15)	0.0202 (5)	
C(4)	0.1122 (3)	0.1453 (7)	−0.0722 (2)	0.0348 (7)	
C(5)	1.1683 (3)	0.6098 (7)	0.31658 (16)	0.0275 (6)	
C(6)	1.1053 (2)	0.6353 (5)	0.39573 (15)	0.0196 (5)	
C(7)	1.1797 (2)	0.8438 (5)	0.43657 (13)	0.0194 (5)	
C(8)	1.4106 (3)	1.0111 (7)	0.46876 (18)	0.0307 (7)	
H(1)	0.5035	0.0467	0.1253	0.020*	
H(2)	0.5528	0.2685	0.0875	0.020*	
H(3)	0.9294	0.8180	0.3643	0.022*	
H(4)	0.9071	0.6773	0.4351	0.022*	
H(5)	0.2210	0.5831	0.1017	0.024*	
H(6)	0.3632	0.5982	0.0508	0.024*	
H(7)	0.2758	0.2067	0.1439	0.020*	
H(8)	0.0229	0.2301	−0.0876	0.042*	
H(9)	0.1869	0.1739	−0.1100	0.042*	
H(10)	0.0921	−0.0254	−0.0691	0.042*	
H(11)	1.2694	0.5498	0.3217	0.033*	
H(12)	1.1712	0.7678	0.2915	0.033*	
H(13)	1.1244	0.4864	0.4257	0.024*	
H(14)	1.5112	0.9920	0.4528	0.037*	
H(15)	1.3707	1.1598	0.4477	0.037*	
H(16)	1.4085	1.0157	0.5249	0.037*	

### Atomic displacement parameters (Å^2^)

**Table:**

	*U*^11^	*U*^22^	*U*^33^	*U*^12^	*U*^13^	*U*^23^
Mo(1)	0.01791 (9)	0.01641 (12)	0.01172 (9)	0.00422 (8)	0.00248 (6)	0.00076 (7)
Mo(2)	0.01801 (10)	0.01769 (12)	0.01263 (9)	0.00569 (8)	0.00168 (6)	0.00010 (7)
S(1)	0.0209 (2)	0.0222 (3)	0.0177 (2)	0.0068 (2)	0.0019 (2)	−0.0043 (2)
S(2)	0.0213 (2)	0.0178 (3)	0.0153 (2)	0.0066 (2)	−0.0001 (2)	−0.0009 (2)
S(3)	0.0215 (2)	0.0160 (3)	0.0164 (2)	0.0027 (2)	−0.0001 (2)	0.0015 (2)
S(4)	0.0215 (2)	0.0456 (5)	0.0204 (2)	0.0023 (3)	0.0050 (2)	−0.0127 (3)
O(1)	0.0243 (9)	0.0227 (11)	0.0174 (8)	0.0032 (8)	0.0043 (7)	0.0019 (7)
O(2)	0.0259 (9)	0.0216 (10)	0.0210 (8)	0.0082 (8)	−0.0017 (7)	0.0028 (7)
O(3)	0.0192 (9)	0.0432 (15)	0.0296 (10)	0.0050 (9)	−0.0040 (7)	−0.0126 (10)
O(4)	0.0265 (9)	0.0281 (12)	0.0171 (8)	0.0035 (8)	0.0029 (7)	−0.0019 (8)
O(5)	0.0188 (8)	0.0347 (13)	0.0230 (9)	0.0061 (8)	−0.0006 (6)	−0.0066 (8)
O(6)	0.0245 (9)	0.0282 (12)	0.0175 (8)	0.0080 (8)	−0.0031 (7)	−0.0018 (8)
N(1)	0.0169 (9)	0.0182 (11)	0.0138 (8)	0.0026 (8)	0.0022 (7)	0.0017 (8)
N(2)	0.0194 (9)	0.0199 (11)	0.0168 (9)	0.0038 (8)	0.0004 (7)	0.0023 (8)
C(1)	0.0177 (11)	0.0198 (13)	0.0222 (12)	0.0027 (9)	−0.0016 (9)	0.0005 (9)
C(2)	0.0157 (10)	0.0189 (13)	0.0161 (10)	0.0017 (9)	0.0016 (8)	0.0001 (9)
C(3)	0.0189 (10)	0.0223 (14)	0.0196 (11)	−0.0022 (10)	0.0025 (8)	0.0015 (9)
C(4)	0.0306 (15)	0.042 (2)	0.0316 (14)	−0.0012 (14)	−0.0089 (12)	−0.0107 (14)
C(5)	0.0210 (11)	0.044 (2)	0.0180 (11)	0.0007 (13)	0.0033 (9)	−0.0086 (12)
C(6)	0.0190 (10)	0.0223 (14)	0.0175 (10)	0.0064 (10)	0.0004 (8)	−0.0008 (9)
C(7)	0.0203 (10)	0.0273 (14)	0.0106 (8)	0.0049 (11)	0.0012 (7)	0.0037 (9)
C(8)	0.0270 (14)	0.041 (2)	0.0244 (13)	−0.0002 (13)	−0.0024 (11)	−0.0038 (13)

### Geometric parameters (Å, °)

**Table:**

Mo(1)—Mo(2)	2.8191 (2)	C(1)—C(2)	1.525 (4)
Mo(1)—S(1)	2.3079 (7)	C(2)—C(3)	1.523 (3)
Mo(1)—S(2)	2.3319 (7)	C(5)—C(6)	1.519 (3)
Mo(1)—S(3)	2.3941 (7)	C(6)—C(7)	1.524 (4)
Mo(1)—O(1)	1.685 (2)	N(1)—H(1)	0.920
Mo(1)—N(1)	2.213 (2)	N(1)—H(2)	0.920
Mo(2)—S(1)	2.3349 (7)	N(2)—H(3)	0.920
Mo(2)—S(2)	2.3028 (7)	N(2)—H(4)	0.920
Mo(2)—S(4)	2.3814 (7)	C(1)—H(5)	0.990
Mo(2)—O(2)	1.693 (2)	C(1)—H(6)	0.990
Mo(2)—N(2)	2.238 (2)	C(2)—H(7)	1.000
S(3)—C(1)	1.826 (2)	C(4)—H(8)	0.980
S(4)—C(5)	1.827 (3)	C(4)—H(9)	0.979
O(3)—C(3)	1.330 (3)	C(4)—H(10)	0.979
O(3)—C(4)	1.458 (4)	C(5)—H(11)	0.990
O(4)—C(3)	1.208 (3)	C(5)—H(12)	0.991
O(5)—C(7)	1.336 (3)	C(6)—H(13)	1.000
O(5)—C(8)	1.453 (4)	C(8)—H(14)	0.980
O(6)—C(7)	1.202 (3)	C(8)—H(15)	0.980
N(1)—C(2)	1.482 (3)	C(8)—H(16)	0.980
N(2)—C(6)	1.482 (3)		
			
S(2)···O(1)^i^	3.405 (2)	H(1)···C(1)^iii^	3.317
S(3)···O(1)^i^	3.445 (2)	H(1)···H(6)^iii^	3.100
S(3)···N(1)^i^	3.218 (2)	H(1)···H(6)^vi^	3.352
S(3)···C(5)^ii^	3.423 (2)	H(1)···H(9)^vi^	3.552
O(1)···S(2)^iii^	3.405 (2)	H(2)···O(4)^x^	2.114
O(1)···S(3)^iii^	3.445 (2)	H(2)···C(3)^x^	3.295
O(1)···O(5)^iv^	3.570 (3)	H(2)···H(6)^vi^	2.727
O(1)···C(8)^iv^	3.278 (3)	H(2)···H(9)^x^	3.320
O(2)···O(5)^v^	3.321 (3)	H(2)···H(10)^x^	3.488
O(2)···O(6)^iii^	3.108 (3)	H(3)···Mo(2)^i^	3.438
O(2)···O(6)^v^	3.217 (3)	H(3)···O(2)^i^	2.404
O(2)···N(2)^iii^	3.165 (3)	H(3)···O(6)^v^	3.556
O(2)···C(7)^v^	3.113 (3)	H(4)···O(2)^i^	3.115
O(4)···N(1)^vi^	2.984 (3)	H(4)···O(6)^v^	2.077
O(4)···C(1)^iii^	3.304 (3)	H(4)···C(7)^v^	3.044
O(4)···C(1)^vi^	3.292 (3)	H(4)···C(8)^v^	3.536
O(4)···C(2)^vi^	3.492 (3)	H(4)···H(13)^viii^	3.006
O(5)···O(1)^vii^	3.570 (3)	H(4)···H(15)^v^	3.319
O(5)···O(2)^viii^	3.321 (3)	H(4)···H(16)^v^	3.139
O(5)···C(8)^ix^	3.334 (4)	H(5)···S(4)^ii^	3.277
O(6)···O(2)^i^	3.108 (3)	H(5)···O(4)^i^	3.397
O(6)···O(2)^viii^	3.217 (3)	H(5)···C(3)^i^	3.545
O(6)···N(2)^viii^	2.903 (3)	H(5)···C(4)^xiii^	3.112
O(6)···C(6)^viii^	3.377 (3)	H(5)···H(8)^xiii^	2.396
N(1)···S(3)^iii^	3.218 (2)	H(5)···H(10)^xiii^	2.982
N(1)···O(4)^x^	2.984 (3)	H(5)···H(12)^ii^	3.512
N(2)···O(2)^i^	3.165 (3)	H(6)···O(4)^i^	2.563
N(2)···O(6)^v^	2.903 (3)	H(6)···O(4)^x^	2.647
C(1)···O(4)^i^	3.304 (3)	H(6)···N(1)^x^	3.421
C(1)···O(4)^x^	3.292 (3)	H(6)···C(3)^i^	3.223
C(2)···O(4)^x^	3.492 (3)	H(6)···C(3)^x^	3.481
C(5)···S(3)^xi^	3.423 (2)	H(6)···H(1)^i^	3.100
C(6)···O(6)^v^	3.377 (3)	H(6)···H(1)^x^	3.352
C(7)···O(2)^viii^	3.113 (3)	H(6)···H(2)^x^	2.727
C(8)···O(1)^vii^	3.278 (3)	H(7)···S(3)^iii^	3.337
C(8)···O(5)^xii^	3.334 (4)	H(7)···S(4)^ii^	3.049
C(8)···C(8)^ix^	3.419 (5)	H(8)···S(4)^vi^	3.533
C(8)···C(8)^xii^	3.419 (5)	H(8)···C(1)^xiv^	3.376
Mo(1)···H(11)^ii^	3.286	H(8)···H(5)^xiv^	2.396
Mo(2)···H(3)^iii^	3.438	H(8)···H(10)^xiii^	3.261
S(1)···H(9)^vi^	3.286	H(9)···S(1)^vi^	3.324
S(1)···H(9)^x^	3.324	H(9)···S(1)^x^	3.286
S(1)···H(10)^x^	3.221	H(9)···H(1)^x^	3.552
S(2)···H(11)^ii^	3.517	H(9)···H(2)^vi^	3.320
S(2)···H(14)^ii^	3.273	H(10)···S(1)^vi^	3.221
S(2)···H(16)^v^	2.945	H(10)···S(4)^vi^	3.543
S(3)···H(1)^i^	2.410	H(10)···O(3)^xiv^	2.985
S(3)···H(7)^i^	3.337	H(10)···H(2)^vi^	3.488
S(3)···H(11)^ii^	2.977	H(10)···H(5)^xiv^	2.982
S(3)···H(12)^ii^	3.125	H(10)···H(8)^xiv^	3.261
S(4)···H(5)^xi^	3.277	H(11)···Mo(1)^xi^	3.286
S(4)···H(7)^xi^	3.049	H(11)···S(2)^xi^	3.517
S(4)···H(8)^x^	3.533	H(11)···S(3)^xi^	2.977
S(4)···H(10)^x^	3.543	H(11)···O(1)^xi^	2.753
O(1)···H(11)^ii^	2.753	H(11)···H(15)^iii^	3.222
O(1)···H(12)^iv^	3.504	H(12)···S(3)^xi^	3.125
O(1)···H(14)^iv^	3.039	H(12)···O(1)^vii^	3.504
O(1)···H(15)^iv^	2.864	H(12)···H(5)^xi^	3.512
O(2)···H(3)^iii^	2.404	H(13)···O(2)^viii^	3.426
O(2)···H(4)^iii^	3.115	H(13)···O(6)^iii^	2.693
O(2)···H(13)^v^	3.426	H(13)···O(6)^v^	3.056
O(2)···H(14)^iv^	3.280	H(13)···H(4)^v^	3.006
O(2)···H(16)^v^	3.158	H(13)···H(15)^iii^	2.932
O(3)···H(10)^xiii^	2.985	H(14)···S(2)^xi^	3.273
O(4)···H(1)^vi^	3.566	H(14)···O(1)^vii^	3.039
O(4)···H(2)^vi^	2.114	H(14)···O(2)^vii^	3.280
O(4)···H(5)^iii^	3.397	H(14)···O(5)^xii^	2.974
O(4)···H(6)^iii^	2.563	H(14)···C(8)^ix^	3.105
O(4)···H(6)^vi^	2.647	H(14)···C(8)^xii^	3.294
O(5)···H(14)^ix^	2.974	H(14)···H(14)^ix^	3.267
O(5)···H(15)^ix^	3.480	H(14)···H(14)^xii^	3.267
O(5)···H(16)^ix^	3.020	H(14)···H(15)^ix^	2.751
O(6)···H(3)^viii^	3.556	H(14)···H(16)^ix^	2.802
O(6)···H(4)^viii^	2.077	H(14)···H(16)^xii^	3.057
O(6)···H(13)^i^	2.693	H(15)···O(1)^vii^	2.864
O(6)···H(13)^viii^	3.056	H(15)···O(5)^xii^	3.480
N(1)···H(6)^vi^	3.421	H(15)···C(8)^xii^	3.146
C(1)···H(1)^i^	3.317	H(15)···H(4)^viii^	3.319
C(1)···H(8)^xiii^	3.376	H(15)···H(11)^i^	3.222
C(3)···H(2)^vi^	3.295	H(15)···H(13)^i^	2.932
C(3)···H(5)^iii^	3.545	H(15)···H(14)^xii^	2.751
C(3)···H(6)^iii^	3.223	H(15)···H(16)^xii^	2.881
C(3)···H(6)^vi^	3.481	H(16)···S(2)^viii^	2.945
C(4)···H(5)^xiv^	3.112	H(16)···O(2)^viii^	3.158
C(7)···H(4)^viii^	3.044	H(16)···O(5)^xii^	3.020
C(8)···H(4)^viii^	3.536	H(16)···C(8)^ix^	3.289
C(8)···H(14)^ix^	3.294	H(16)···C(8)^xii^	3.245
C(8)···H(14)^xii^	3.105	H(16)···H(4)^viii^	3.139
C(8)···H(15)^ix^	3.146	H(16)···H(14)^ix^	3.057
C(8)···H(16)^ix^	3.245	H(16)···H(14)^xii^	2.802
C(8)···H(16)^xii^	3.289	H(16)···H(15)^ix^	2.881
H(1)···S(3)^iii^	2.410	H(16)···H(16)^ix^	3.402
H(1)···O(4)^x^	3.566	H(16)···H(16)^xii^	3.402
			
Mo(2)—Mo(1)—S(1)	53.049 (18)	S(4)—C(5)—C(6)	111.2 (2)
Mo(2)—Mo(1)—S(2)	52.068 (17)	N(2)—C(6)—C(5)	108.9 (2)
Mo(2)—Mo(1)—S(3)	126.612 (19)	N(2)—C(6)—C(7)	111.5 (2)
Mo(2)—Mo(1)—O(1)	106.05 (6)	C(5)—C(6)—C(7)	108.6 (2)
Mo(2)—Mo(1)—N(1)	133.81 (5)	O(5)—C(7)—O(6)	124.7 (2)
S(1)—Mo(1)—S(2)	101.77 (2)	O(5)—C(7)—C(6)	110.6 (2)
S(1)—Mo(1)—S(3)	133.33 (2)	O(6)—C(7)—C(6)	124.6 (2)
S(1)—Mo(1)—O(1)	111.82 (7)	Mo(1)—N(1)—H(1)	109.1
S(1)—Mo(1)—N(1)	81.45 (6)	Mo(1)—N(1)—H(2)	109.1
S(2)—Mo(1)—S(3)	81.17 (2)	C(2)—N(1)—H(1)	109.1
S(2)—Mo(1)—O(1)	106.77 (7)	C(2)—N(1)—H(2)	109.2
S(2)—Mo(1)—N(1)	152.22 (6)	H(1)—N(1)—H(2)	107.9
S(3)—Mo(1)—O(1)	111.63 (7)	Mo(2)—N(2)—H(3)	108.8
S(3)—Mo(1)—N(1)	77.05 (6)	Mo(2)—N(2)—H(4)	108.8
O(1)—Mo(1)—N(1)	97.23 (9)	C(6)—N(2)—H(3)	108.8
Mo(1)—Mo(2)—S(1)	52.177 (17)	C(6)—N(2)—H(4)	108.8
Mo(1)—Mo(2)—S(2)	53.005 (18)	H(3)—N(2)—H(4)	107.7
Mo(1)—Mo(2)—S(4)	123.814 (18)	S(3)—C(1)—H(5)	109.6
Mo(1)—Mo(2)—O(2)	105.19 (7)	S(3)—C(1)—H(6)	109.6
Mo(1)—Mo(2)—N(2)	135.42 (6)	C(2)—C(1)—H(5)	109.6
S(1)—Mo(2)—S(2)	101.83 (2)	C(2)—C(1)—H(6)	109.6
S(1)—Mo(2)—S(4)	79.91 (2)	H(5)—C(1)—H(6)	108.1
S(1)—Mo(2)—O(2)	105.39 (7)	N(1)—C(2)—H(7)	108.7
S(1)—Mo(2)—N(2)	155.64 (6)	C(1)—C(2)—H(7)	108.7
S(2)—Mo(2)—S(4)	129.80 (3)	C(3)—C(2)—H(7)	108.7
S(2)—Mo(2)—O(2)	112.22 (7)	O(3)—C(4)—H(8)	109.4
S(2)—Mo(2)—N(2)	82.64 (6)	O(3)—C(4)—H(9)	109.4
S(4)—Mo(2)—O(2)	115.52 (7)	O(3)—C(4)—H(10)	109.4
S(4)—Mo(2)—N(2)	79.03 (6)	H(8)—C(4)—H(9)	109.5
O(2)—Mo(2)—N(2)	94.67 (9)	H(8)—C(4)—H(10)	109.5
Mo(1)—S(1)—Mo(2)	74.77 (2)	H(9)—C(4)—H(10)	109.6
Mo(1)—S(2)—Mo(2)	74.93 (2)	S(4)—C(5)—H(11)	109.4
Mo(1)—S(3)—C(1)	104.40 (10)	S(4)—C(5)—H(12)	109.4
Mo(2)—S(4)—C(5)	103.96 (9)	C(6)—C(5)—H(11)	109.4
C(3)—O(3)—C(4)	116.4 (2)	C(6)—C(5)—H(12)	109.4
C(7)—O(5)—C(8)	116.6 (2)	H(11)—C(5)—H(12)	108.0
Mo(1)—N(1)—C(2)	112.43 (15)	N(2)—C(6)—H(13)	109.3
Mo(2)—N(2)—C(6)	113.75 (18)	C(5)—C(6)—H(13)	109.2
S(3)—C(1)—C(2)	110.37 (18)	C(7)—C(6)—H(13)	109.3
N(1)—C(2)—C(1)	108.5 (2)	O(5)—C(8)—H(14)	109.4
N(1)—C(2)—C(3)	111.0 (2)	O(5)—C(8)—H(15)	109.5
C(1)—C(2)—C(3)	111.0 (2)	O(5)—C(8)—H(16)	109.5
O(3)—C(3)—O(4)	124.2 (2)	H(14)—C(8)—H(15)	109.5
O(3)—C(3)—C(2)	111.6 (2)	H(14)—C(8)—H(16)	109.4
O(4)—C(3)—C(2)	124.2 (2)	H(15)—C(8)—H(16)	109.5

Symmetry codes: (i) *x*, *y*+1, *z*; (ii) *x*−1, *y*, *z*; (iii) *x*, *y*−1, *z*; (iv) *x*−1, *y*−1, *z*; (v) −*x*+2, *y*−1/2, −*z*+1; (vi) −*x*+1, *y*−1/2, −*z*; (vii) *x*+1, *y*+1, *z*; (viii) −*x*+2, *y*+1/2, −*z*+1; (ix) −*x*+3, *y*−1/2, −*z*+1; (x) −*x*+1, *y*+1/2, −*z*; (xi) *x*+1, *y*, *z*; (xii) −*x*+3, *y*+1/2, −*z*+1; (xiii) −*x*, *y*+1/2, −*z*; (xiv) −*x*, *y*−1/2, −*z*.

### Hydrogen-bond geometry (Å, °)

**Table:**

*D*—H···*A*	*D*—H	H···*A*	*D*···*A*	*D*—H···*A*
N(1)—H(2)···O(4)^x^	0.92	2.11	2.984 (3)	157
N(2)—H(3)···O(2)^i^	0.92	2.40	3.165 (3)	140
N(2)—H(4)···O(6)^v^	0.92	2.08	2.903 (3)	149

Symmetry codes: (x) −*x*+1, *y*+1/2, −*z*; (i) *x*, *y*+1, *z*; (v) −*x*+2, *y*−1/2, −*z*+1.
